# Refinement of the acute inhalation limit test for inert, nano-sized dusts by an *in silico* dosimetry-based evaluation: case study for the dissolution of a regulatory dilemma

**DOI:** 10.3389/ftox.2023.1258861

**Published:** 2023-12-05

**Authors:** Heidi Stratmann, Lan Ma-Hock, Simone Tangermann, Richard A. Corley

**Affiliations:** ^1^ Colors and Effects Switzerland AG Efringerstrasse, Basel, Switzerland; ^2^ Experimental Toxicology and Ecology, BASF SE, Ludwigshafen, Germany; ^3^ Greek Creek Toxicokinetics Consulting, LLC, Boise, ID, United States

**Keywords:** MPPD, aerosol deposition, acute inhalation testing, REACH, GHS, nanomaterial, modeling, dust

## Abstract

This case study aims to describe the dilemma faced when exposing rats to very high concentrations of fine, pulverulent materials for acute inhalation studies and to address the regulatory question of whether the effects seen here are relevant to humans and the subject of classification according to the Globally Harmonized System of Classification and Labeling of Chemicals (GHS). Many powders match the definition of nanomaterials in the EU; therefore, information on acute inhalation testing of powders up to the GHS cutoff of 5 mg/L is required. However, testing rats at such a high aerosol concentration can cause physical obstruction of the airways and even mortality by suffocation. Therefore, to evaluate whether the physical effects on airway obstruction in rats exposed to 5 mg/L for 4 hours and alternative exposures to 1 and 2 mg/L are relevant for humans, an *in silico* evaluation of aerosol deposition was conducted using the multiple-path particle dosimetry (MPPD) model. For this evaluation, actual exposure conditions for an organic, nano-sized pigment which produced 100% lethality in rats at 5 mg/L, but not at 1 mg/L, were used to assess the potential for airway obstruction in rats and accordingly in humans. As an indicator of the potential for airway obstruction, the ratio of the diameter of the deposited, aggregated aerosol to airway diameter was calculated for each exposure condition. For rats exposed to 5 mg/L for 4 h, approximately 75% of tracheobronchial and 22% of pulmonary/alveolar airways were considered vulnerable to significant or complete obstruction (ratios >0.5). In humans, an equivalent exposure resulted in just over 96% of human tracheobronchial airways that received deposited mass to airway diameter ratios between 0.3 and 0.4 (nasal) or 0.4 and 0.5 (oral), with no airways with ratios >0.5. For the pulmonary/alveolar region, ∼88% of the airways following nasal or oral breathing were predicted to have deposited aerosol diameter to airway diameter ratios <0.1, with no airways with ratios >0.5. Thus, the *in silico* results obtained for rats are in line with the pathological findings of the animal test. The predicted results in humans, however, affirm the hypothesis of a rat-specific high dose effect which does not justify a classification according to GHS.

## 1 Introduction

When REACH came into force in 2007, producers and importers of chemicals in Europe began to register their substances according to this new regulation. One of the key points of REACH is “no data, no market.” Thus, affected companies collected and evaluated existing experimental data required for the registration deadlines, with the relevant deadline depending on the tonnage. The REACH regulation was amended in 2020 by the notification obligation for nanomaterials. With this amendment, acute inhalation testing was no longer an option depending on the most likely route of exposure but a mandatory information requirement even for low-volume substances.

For poorly soluble particles that are either known or expected to be virtually non-toxic, the current OECD guidelines for acute inhalation toxicity assessments incorporate a limit test concentration for aerosols (OECD, 2009). Depending upon the regulatory requirements, the limit test concentration for aerosols can be as high as 5 mg/L (or the maximum attainable concentration) if the United Nations (UN) Globally Harmonized System of Classification and Labeling of Chemicals (GHS) is applied. The OECD guidance document for inhalation toxicity testing (OECD, 2018) points out that the GHS limit concentration of 5 mg/L is technically challenging for most aerosols and exceeds real-world human exposure. A maximum aerosol concentration of 2 mg/L is therefore recommended. However, according to the GHS, an exposure concentration below 5 mg/L is a criterion for placement in hazard category 4 and labeling as “harmful after inhalation,” even if no mortality was observed. Thus, the regulatory thresholds for classification and labeling are at this point in contradiction with the recommendations of the OECD guidelines. Eventually, the registrant of a nanomaterial is forced to weigh the OECD recommendations and animal welfare against a possible erroneous classification for product labeling.

The aim of this case study is to examine whether *in vivo* effects of obstructed airway-induced lethality observed in a recently conducted, regulatory-required acute inhalation toxicity study in rats with an organic, nano-sized pigment (Wittmer et al., 2021) are relevant for toxicity classification and labeling. Similar findings of airway obstruction in rats following acute inhalation exposures have also been observed in the past for other organic, nano-sized pigments (Hofmann et al., 2018). This latest toxicity study by Wittmer et al. thus prompted the need for additional evaluations beyond those by Hoffmann et al. regarding the importance of physicochemical properties in causing airway obstruction to include an *in silico* analysis of aerosol dosimetry. We therefore used the well-established multiple-path particle dosimetry (MPPD) model to provide a respiratory dosimetry assessment of the potential for airway obstruction following *in vivo* exposures of rats and humans to aerosols under the relevant aerosol exposure conditions used in Wittmer et al. (2021).

It is widely recognized that external exposure concentrations of airborne particulates are not directly equivalent to the amount that is inhaled, deposited, and retained in airways of laboratory animals *versus* humans due to major differences in airway anatomy and physiology (c.f. Menache et al., 1995; Werner and Asgharian, 2003; Tawhai et al., 2004; Brown et al., 2005; Phalen and Mendez, 2009; Miller et al., 2016; Bevan et al., 2018; Corley et al., 2021). Without factoring in these differences across species, the results from inhalation toxicity studies may not be directly translatable to human health risk. As a result, computational approaches such as MPPD that combine species-specific data and the physics of aerosol transport and deposition are increasingly utilized by regulatory agencies to carry out their assessments (c.f. EPA, 2021).

The purpose of our computational approach using MPPD is to provide a generic aerosol respiratory dosimetry assessment tool for calculating the potential for airway obstruction in rats using the physical attributes of aerosol exposure (e.g., concentration, size, density, and duration) from the acute toxicity case study. Emphasis was placed on estimations of particle load in individual airways following multiple acute exposures to assess the potential for physical obstruction of bronchial and terminal pulmonary airways in rats due to high rates of local aerosol deposition, and the resulting challenges for study interpretation and product classification and labeling. For comparison, human simulations for 5 mg/L exposure were also conducted to highlight cross-species differences in regional and airway-specific deposition where lethality was observed in rats. In this way, we hope to encourage future action to resolve the discrepancies between regulatory classification and guidelines for acute inhalation toxicity testing requirements and thereby reduce unnecessary testing in laboratory animals, especially when airway obstruction-induced lethality can be anticipated.

## 2 Materials and methods

### 2.1 Test material

An organic pigment, CAS 27614-71-7, was used as the test item in the acute inhalation toxicity study in rats. The sample is handled as blue powder (median D50 14.9 nm) having a purity of >99%. Although the test material mainly consisted of nano-sized particles, the need to comply with the high atmospheric concentration stipulated by the OECD test guideline 403 limit test led to substantial and inevitable agglomeration of particles. As a result, the test atmosphere contained micro-sized agglomerates/particles instead (Wittmer et al., 2021).

### 2.2 Acute inhalation study

According to OECD guideline 403*,* male and female Wistar rats were exposed by inhalation to dust atmosphere of the test substance in two sequential experiments at gravimetrically determined concentrations of 5.212 mg/L and 1.084 mg/L. No mortality was observed at 1.084 mg/L, but all animals exposed to 5.212 mg/L died either during exposure or on study days 1–2 post-exposure. Therefore, gross pathological examinations were performed on all animals, and histological examinations of the respiratory tract were performed in three representative dead male and female rats (Wittmer et al., 2021). A summary of the study design, exposure generation and characterization, and results are provided in the Supplementary Material.

### 2.3 Choice of the *in silico* model

The MPPD model developed for calculating the deposition and clearance of inhaled insoluble particles was utilized for this dosimetry-based analysis of the limit test exposure conditions for poorly soluble, low-toxicity materials to meet the regulatory requirements of GHS (OECD, 2009). The MPPD model was chosen as it is a mechanistic model that combines many of the complexities of species-specific anatomy, physiology, and aerosol physics in an easy-to-use platform for personal computers.

Two versions of the model were available and utilized for this project: (a) the public version (v3.041), which can be obtained freely from Applied Research Associates (ARA; https://www.ara.com/mppd/), and (b) a modification of the public version obtained from ARA (v 3.24C57) that fixed an issue with the asymmetric Sprague–Dawley rat airway geometry used in the model for the head region.

### 2.4 MPPD input conditions for aerosol dosimetry in rats

While the acute inhalation test case study was carried out on Wistar rats, the MPPD model is currently configured only for Sprague–Dawley and Long Evans rat strains. Due to their age- and body weight-dependent anatomic similarities, the MPPD model for Sprague–Dawley rats was used to determine regional and airway-specific aerosol deposition representative of a guideline limit test acute inhalation toxicity study (OECD, 2009). Specifically, a 4-hr nose-only inhalation exposure to aerosol particles with mass median aerosol diameters (MMADs), geometric standard deviations (GSDs), and aerosol concentrations from the Wittmer et al. acute toxicity study were simulated along with an additional aerosol concentration (2 mg/L) that is recommended for an alternative limit test. For rats simulations, breathing physiology used MPPD’s default, resting physiology for a male Sprague–Dawley rat adjusted for a body weight of 300 g was used (Table 1). Simulations were not performed for female Sprague–Dawley rats as results would be similar as MPPD provides for adjustments in anatomy and physiology for the lower body weights (Miller et al., 2014). Since the inhalability of aerosol particles in rats decreases for particles >1 µm MMAD (Ménache et al., 1995), inhalability correction was used in all rat simulations.

**TABLE 1:** Species, physiology, and aerosol properties used in each MPPD simulation.

		Physiology				Aerosol properties				
Species	Position	Breathing modality	Activity	Tidal Vol. (ml/breath)	Frequency (BPM)	Conc. (mg/m^3^)	MMAD (µm)	GSD	Density (g/cm^3^)	Inhalability adjustment (yes/no)
SD rat, asymmetric, 300 g BW	Upright	Nose only	Resting	2.12614 (scaled by BW)	166	5212^a^	2.74^a^	2.8^a^	1.0	Yes
SD rat, asymmetric, 300 g BW	Upright	Nose only	Resting	2.12614 (scaled by BW)	166	2000^b^	2.01	3.0	1.0	Yes
SD rat, asymmetric, 300 g BW	Upright	Nose only	Resting	2.12614 (scaled by BW)	166	1084^a^	2.01^a^	3.0^a^	1.0	Yes
Human, Yeh and Schum, symmetric	Upright	Nasal	Resting	625 (default)	12 (default)	5212	2.74	2.8	1.0	No
Human, Yeh and Schum, symmetric	Upright	Oral	Resting	625 (default)	12 (default)	5212	2.74	2.8	1.0	No

^a^ Exposure conditions from Wittmer et al. (2021).

^b^ OECD, recommended alternate limit test exposure concentration (OECD, 2009).

The asymmetric (multiple-path) Sprague–Dawley rat model in MPPD was used to provide a detailed (airway-specific) analysis of the heterogeneity of aerosol deposition across the lungs. This feature (asymmetry) is particularly important for rats due to their monopodial branching patterns, where a significant number of respiratory bronchioles and alveolar regions branch off from early generations of conducting airways, unlike the symmetric model included in MPPD where terminal alveolar regions are represented only in the final eight generations of each tracheobronchial conducting airway.

### 2.5 MPPD input conditions for aerosol dosimetry in humans

Human simulations were conducted under the same aerosol exposure conditions used for rats simulation (high concentration) as this was the only concentration associated with mortality in the study of acute inhalation exposures in rats. Since humans are not obligate nose-breathers like rats and potential human exposures to many aerosols may occur under both residential and occupational exposure conditions, both nasal and oral breathing were simulated under resting conditions (ICRP, 1994) (Table 1). The Yeh and Schum symmetric (typical path) model was used for all simulations as this is the recommended default human geometry by the U.S. EPA (EPA, 2021). The inhalability of aerosols in humans decreases for particles >10 µm MMAD (Menache et al., 1995), so inhalability correction was not necessary for these simulations.

### 2.6 Calculating the potential for airway obstruction

In addition to a variety of potential aerosol dose metrics, such as the fraction, mass, particle number, particle surface area, or volume of inhalable particle deposition by region or airway or airway surface area, MPPD also provides airway-segment and/or generation-specific calculations of deposited aerosol volume and mass depending on the choice of the model, exposure conditions, and aerosol properties. As an indicator of the potential for airway obstruction, ratios of the diameter of the deposited, aggregated aerosol, calculated from the volume or mass of the aerosol deposited in each airway, to the diameter of the corresponding airway were calculated for each exposure condition.

## 3 Results

### 3.1 Results of the acute inhalation study

**FIGURE 1:**
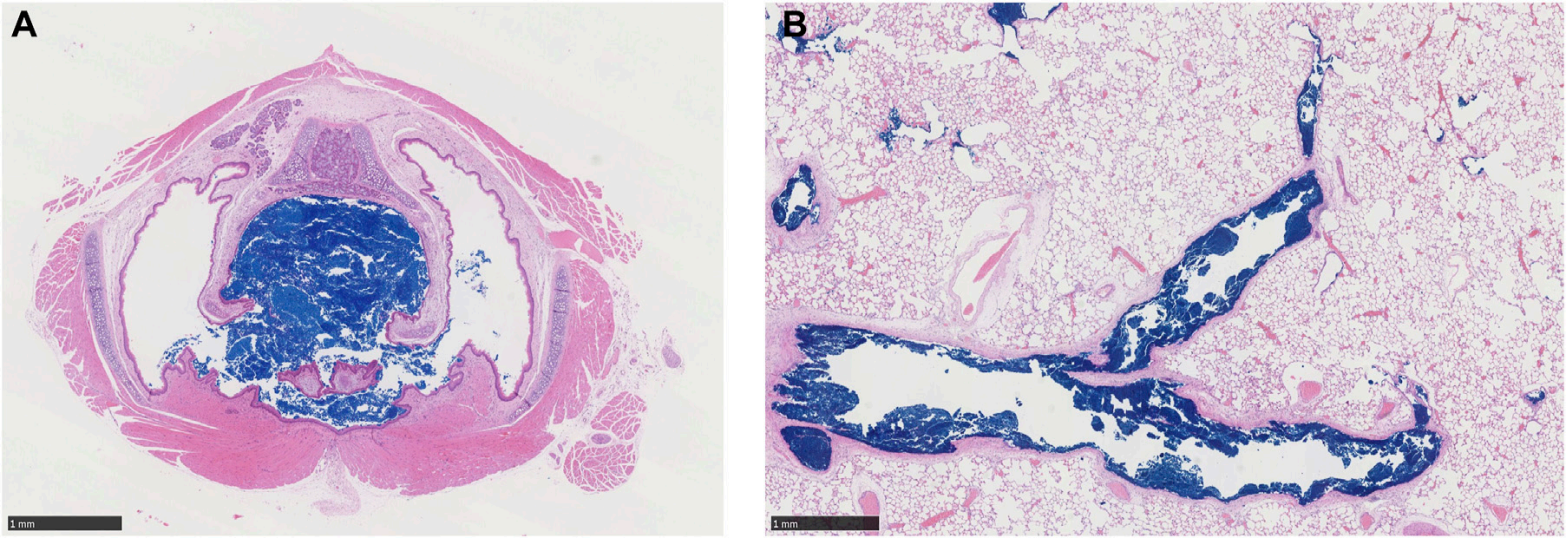
Representative histology sections (H&E stain, ×20 magnification, black bar scale of 1 mm in each panel) through **(A)** larynx level 1 and **(B)** lungs of a male rat showing accumulation of deposited copper phthalocyanine pigment (dark blue) in respiratory airways and complete obstruction of the larynx during an acute, 4-h exposure to 5.212 mg/L aerosol (Wittmer et al., 2021).

Cascade impactor measurements resulted in mass median aerodynamic diameters (MMADs) between 1.93 and 2.98 µm, with high fractions of particles < MMAD 3 µm. The particles were larger at high concentration than at low concentration, which was unavoidable due to agglomeration. When exposed to 5.212 mg/L, all animals died either during exposure or during the first 2 days after the exposure, while no animals died when exposed to 1.084 mg/m³ (Wittmer et al., 2021). Clinical signs of toxicity comprised accelerated, abdominal respiration and respiration sounds of different severity patterns and durations, indicating impairment of respiration. There were no substance-related gross pathological findings in the surviving animals at the end of the post-exposure observation period (day 14). In animals that died, many black foci were seen in the lung lobes with a sunken surface. Blue discoloration of the content of the stomach was seen in four males and three females, and blue depositions in the trachea were present in four males and two females (Supplementary Table S1.2). There were no gross pathological changes in the remote organs of these prematurely dead animals. Since this was a guideline toxicity study, histological examinations were carried out only on rats that died during exposure or during the 2-week post-exposure observation period. These examinations revealed that large amounts of blue pigment were deposited in the larynx, bronchi, bronchioles, and terminal bronchioles, causing obstruction of the airway and subsequently leading to death (Figure 1 and Supplementary Table S1.3). In the nasal cavity, small to moderate amounts of the blue pigment were observed. Based on the lethality rate, clinical signs of toxicity, body weight development, and gross necropsy, there were no differences in response between male and female animals.

### 3.2 *In silico* aerosol deposition in rats

**FIGURE 2:**
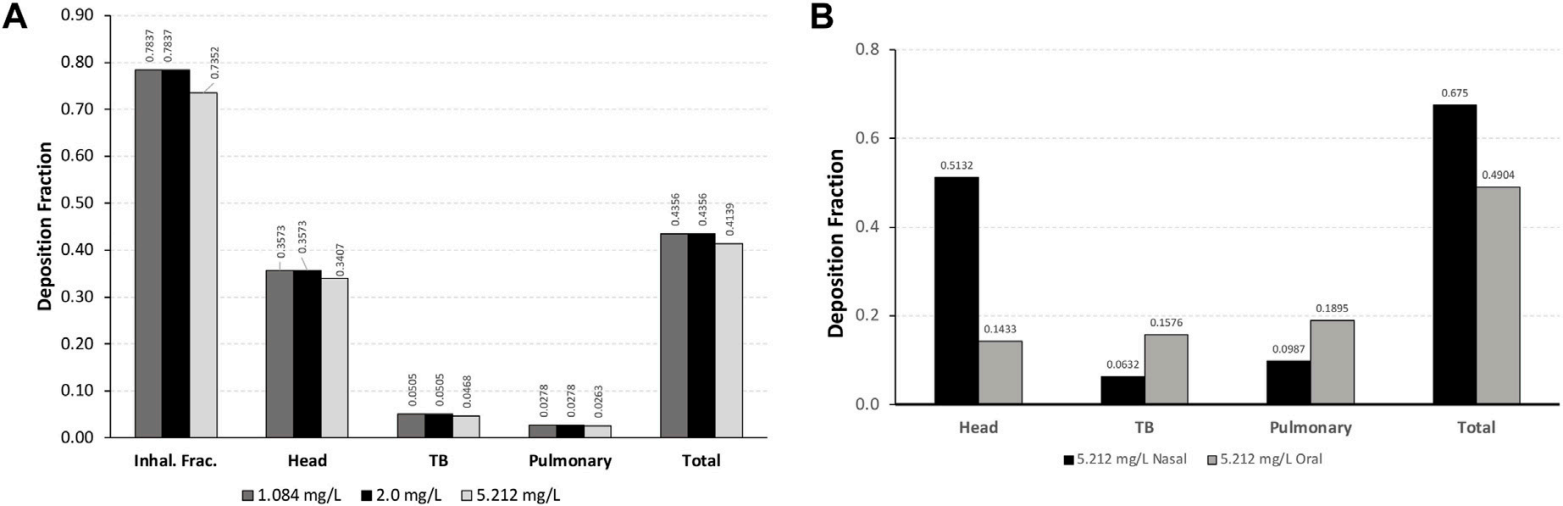
Comparisons of the inhalable fraction (rat only) and deposition fractions of aerosol particles in each region of **(A)** a asymmetric Sprague–Dawley rat following a 4-h exposure to aerosol concentrations of 1.084, 2.0, and 5.212 mg/L and **(B)** Yeh and Schum symmetric human models following nasal and oral breathing at 5.212 mg/L under resting ventilation conditions. For human simulations, the inhalable fraction of particle sizes at 5.212 mg/L was 1.0 (see Table 1 for simulation details).

**FIGURE 3:**
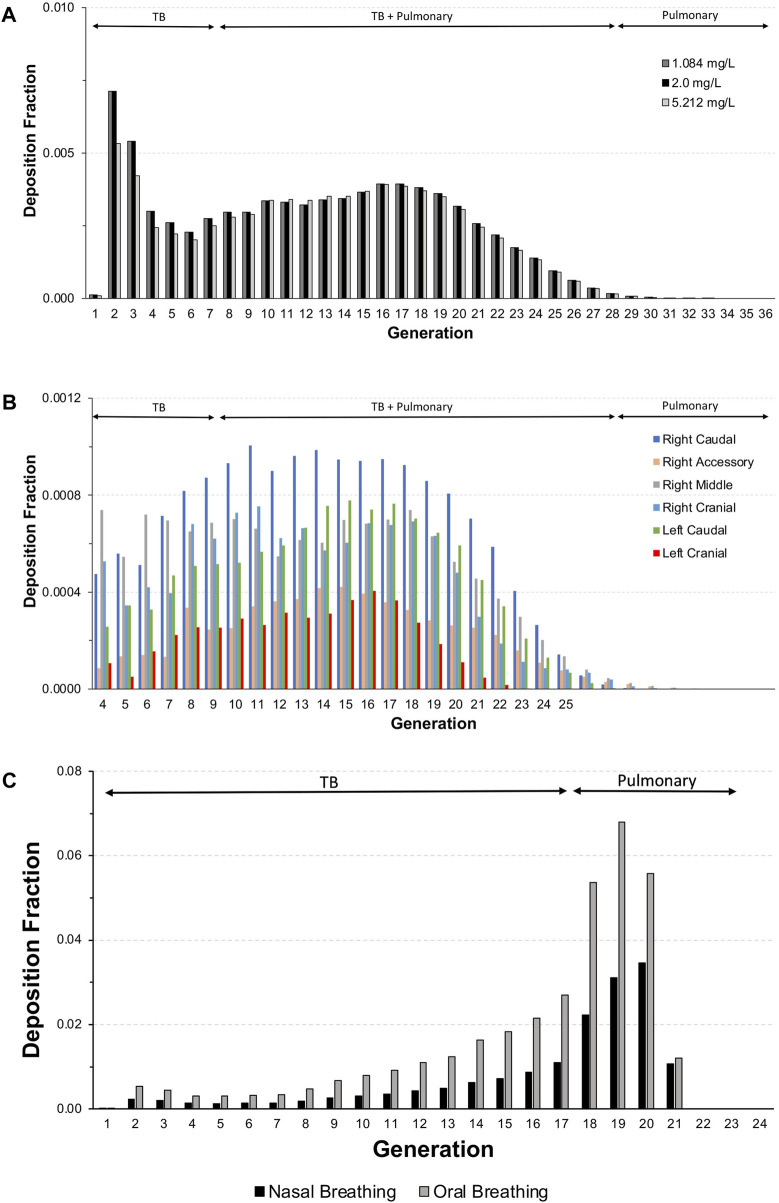
Deposition fractions in each generation of the lung (sum of all tracheobronchial and pulmonary airways in each generation) in **(A)** the entire lung and **(B)** individual lobes of the asymmetric Sprague–Dawley rat model following a 4-h exposure to aerosol concentrations of 1.084, 2.0, and 5.212 mg/L and **(C)** the Yeh and Schum human model following nasal and oral breathing (5.212 mg/L only) under resting ventilation conditions (see Table 1 for simulation details).

For each exposure, 41–44% of the total inhalable particles deposited in the head (∼34–35%; nose through larynx/throat), conducting airways in the chest (∼5%; tracheobronchial, TB), and pulmonary/alveolar (∼3%) regions in the Asymmetric rat models (Figure 2A). In the chest region in the asymmetric Sprague–Dawley rat model, tracheobronchial airways cover the first 28 generations (generation 1 representing the trachea), with the pulmonary airways branching off of the tracheobronchial airways from generations 8 to 28 and an additional eight generations terminating each complete tracheobronchial airway for a total of 36 generations (Figure 2A. Airway generation-specific deposition over the entire lung is shown in Figure 3A. However, the distribution of particles deposited at each generation varied up to an order of magnitude between the lobes (Figure 3B), reflecting the realistic monopodial airway branching and geometry measured from lung casts used in the MPPD asymmetric Sprague–Dawley rat model (Schroeter et al., 2012; Miller et al., 2014).

### 3.3 Potential for obstructive disruption of airway function in rats

While MPPD is not designed to specifically address the question of whether deposited aerosols can be of sufficient magnitude to alter local airflows and thus *pulmonary* function (i.e., gas exchange), the significant amounts of aerosol that deposit in individual tracheobronchial and pulmonary regions of the asymmetric Sprague–Dawley rat simulations suggest that the potential for disruption in airflows and lung function is possible or even likely. To assess this possibility, the total mass and volume of aerosol particles deposited in each tracheobronchial and pulmonary airway over the 4-h exposure were assumed to coalesce into a single spherical volume whose dimensions (diameter) were then compared with each corresponding airway diameter. While MPPD has no equations describing the influence of particles on airflows, significant deposition and accumulation certainly occurs in discrete regions within each airway over a 4-h exposure. This comparative calculation from MPPD currently ignores this potential effect on local airflow that would enhance deposition in proximal airways rather than allowing aerosols to penetrate distally without obstruction.

**FIGURE 4:**
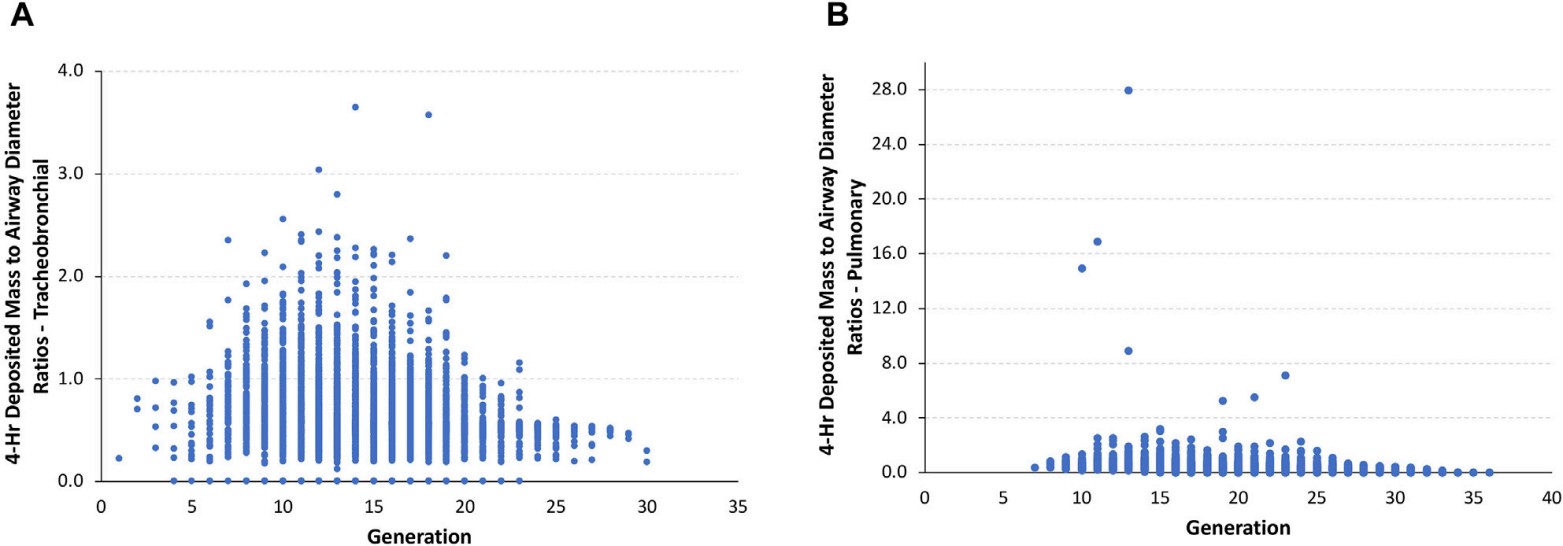
Ratios of the theoretical diameters of retained particle mass (volume) to their corresponding airway diameters in **(A)** tracheobronchial and **(B)** pulmonary airways within each generation of the asymmetric Sprague–Dawley rat models following a 4-h exposure to a limit test concentration of 5.212 mg/L aerosol.

Given this scenario, the ratios of the theoretical diameter of the total deposited mass in each airway to its corresponding airway diameter in the asymmetric Sprague–Dawley rat simulations were determined at the end of the 4-h exposure. In this model, the ratios of deposited aerosol volume diameters to the corresponding diameter of each airway in the tracheobronchial and pulmonary regions show a significant number of airways with ratios >0.5 and, in many cases, ratios significantly >1.0 (fully blocked) after exposure to 5.212 mg/L (Figure 4).

**FIGURE 5:**
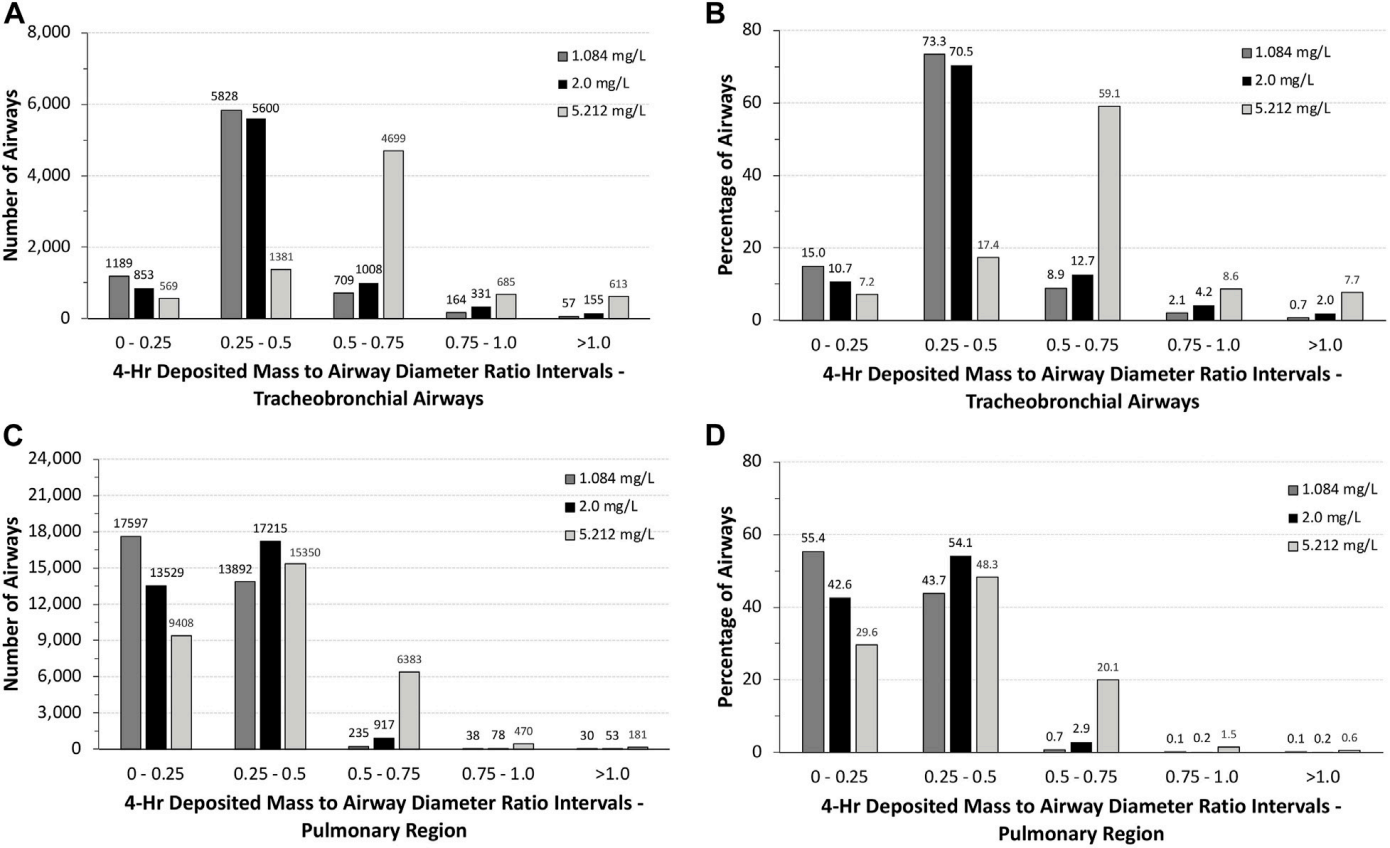
Distributions of the total numbers and percentages of the ratios of theoretical diameters of deposited particle mass (volume) to their corresponding airway diameters in all **(A,B)** tracheobronchial and **(C,D)** pulmonary airways of the asymmetric Sprague–Dawley rat model following a 4-h exposure to 1.084, 2.0, and 5.212 mg/L aerosol concentrations. Ratios are grouped in intervals of 0–0.25, 0.25–0.5, 0.5–0.75, 0.75–1.0, and >1.0.

The total number of airways and the percentage of airways were sorted into quartile intervals of the ratio of deposited volume to airway diameter ranging from 0 to 0.25, 0.25 to 0.5, 0.5 to 0.75, 0.75 to 1.0, and >1.0 to gauge the relative potential for biologically significant airway obstruction following exposures to 1.084 and 5.212 mg/L of aerosols and the alternative limit test concentration of 2 mg/L (Figure 5). At the highest exposure, 5.212 mg/L, with 100% mortality in the acute inhalation study, most airways in the tracheobronchial region (4,699 airways or 59.1%) had diameter ratios between 0.5 and 0.75, 8.6% (685 airways) had ratios between 0.75 and 1.0, and 7.7% of airways (613 airways) had ratios >1.0. In the pulmonary region, the number and percentages of airways having ratios >0.5 were smaller than that predicted for the tracheobronchial region, at just over 22% (7,034 airways). However, there were numerous airways with deposited aerosol volume to airway diameter ratios significantly greater than 1.0, some with ratios up to 28. These high ratios are consistent with the number of airways that branch off from the upper bronchiolar airways (Miller et al., 2014), indicating greater exposure potential for aerosols in these locations (Figure 4).

At the lowest concentration used in the acute inhalation toxicity study (1.084 mg/L) with no mortality and at 2 mg/L, which has been suggested by OECD as an alternative limit test concentration, there was a shift toward greater numbers and percentages of airways with ratios of deposited aerosol volume to airway diameters <0.5 in both tracheobronchial and pulmonary airways (Figure 5). While the number and percentages of airways with ratios >1 also significantly decreased at the two lower exposure concentrations vs. 5.212 mg/L, there were still a significant percentage of airways with ratios >0.5 in both tracheobronchial and pulmonary airways combined (∼3 to 6%), 87–208 airways (mostly tracheobronchial) with ratios >1.0, and some airways with ratios nearly 18-fold greater than 1.0, indicating that some airways may yet be compromised by physical obstruction at lower exposure concentrations even if mortality did not occur at the lowest concentration (1.084 mg/L) in the acute inhalation study (the alternative limit test concentration of 2 mg/L was not tested in the acute inhalation study).

### 3.4 Aerosol dosimetry in humans

Following nasal breathing, 51.3% of the inhaled fraction of aerosols deposited in the head, while 6.3% deposited in the tracheobronchial and 9.9% in the pulmonary regions (68% total; Figure 2B). This is in contrast with oral breathing, where under resting conditions only 14.3% of the inhaled fraction of aerosols deposited in the head, with deposition increasing to 15.8% in the tracheobronchial and 19% in the pulmonary regions (49% total).

For the chest region, the symmetric Yeh and Schum human model consists of 17 generations of tracheobronchial airways (the trachea is generation 1), with seven generations of pulmonary airways appended to each terminal bronchiole airway for a total of 24 generations. In contrast to rats model, generation-specific deposition fractions for each breathing modality, especially oral breathing, indicate a significant shift in fractional deposition from the upper conducting airways toward the deeper pulmonary airways in generations 18–21 (Figure 3C). However, no deposition was predicted for terminal alveolar generations 22–24, which contain nearly 88% of the airways in the symmetric Yeh and Schum human model. Given the large airway surface in the pulmonary region, the cumulative mass deposited in 4 h per airway surface area remained significantly lower in the pulmonary region compared to the first few generations of the tracheobronchial airways.

### 3.5 Potential for obstructive disruption of airway function in humans

**FIGURE 6:**
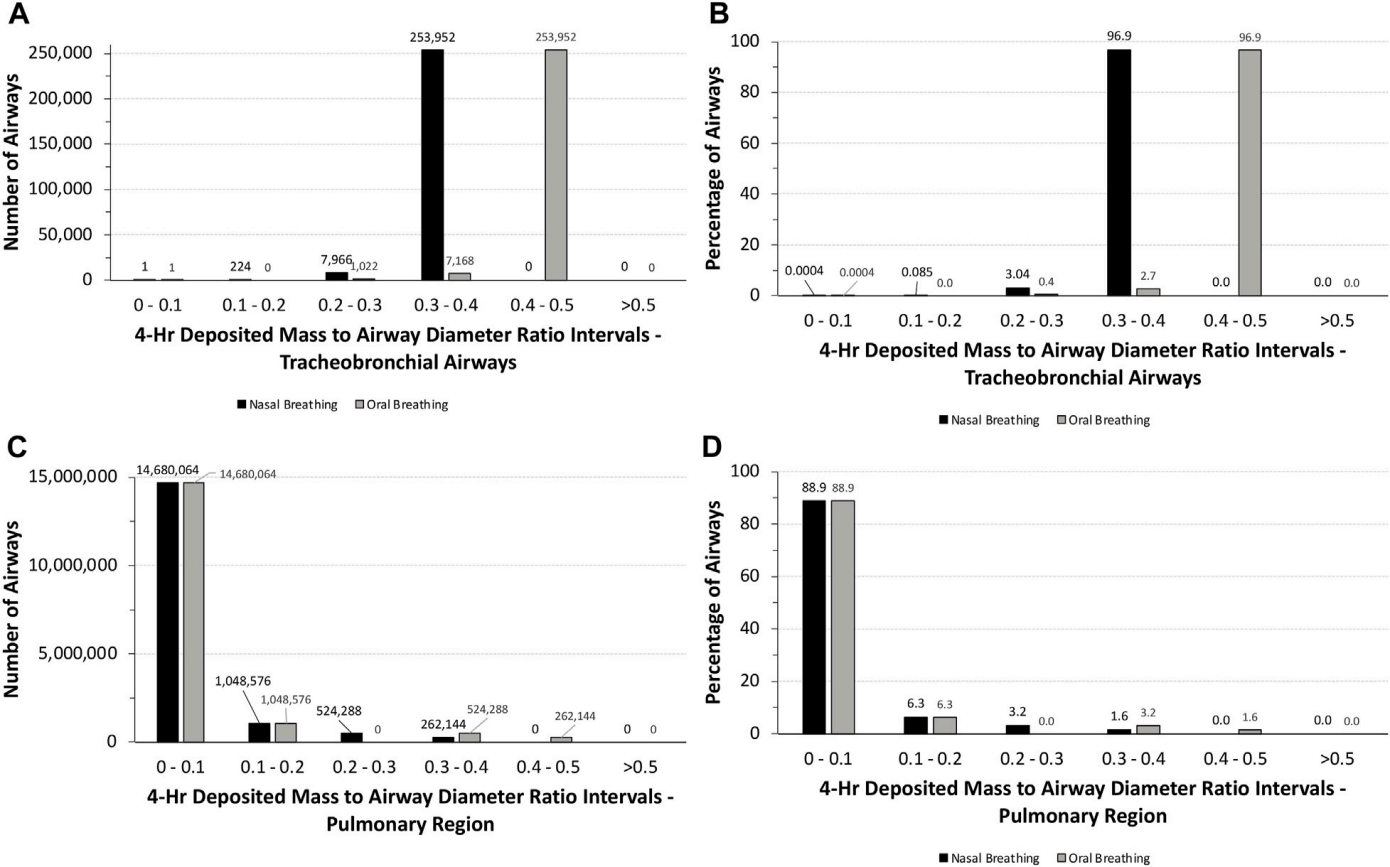
Distributions of the total numbers and percentages of the ratios of theoretical diameters of deposited particle mass (volume) to their corresponding airway diameters in all **(A,B)** tracheobronchial and **(C ,D)** pulmonary airways of the symmetric Yeh and Schum model following a 4-h exposure to 5.212 mg/L aerosol for nasal and oral breathing. Ratios are grouped in intervals of 0–0.1, 0.1–0.2, 0.2–0.3, 0.3–0.4, 0.4–0.5, and >0.5.

Although humans would not be expected to tolerate unrealistic exposures to 5.212 mg/L aerosol concentration for 4 h, a similar analysis of the potential for airway obstruction was used for human simulations following nasal and oral breathing (Table 1). Under these exposure conditions, no airway had theoretical deposited mass to airway diameter ratios >0.5, with nearly 88% of all airways (∼14.7 million) having ratios <0.1 (Figure 6). While the latter number was primarily associated with the pulmonary airways, nearly 790,000 airways (∼4.8%) in the pulmonary region had deposited mass to airway diameter ratios between 0.2 and 0.5 depending on the breathing modality. In the tracheobronchial region, however, nearly 97% (∼254,000) of airways, primarily in generations 13–18, had deposited mass to airway diameter ratios between 0.3 and 0.4 (nasal) or 0.4 and 0.5 (oral), showing the shift in penetration to deeper airways following oral breathing. Thus, there may be some degree of potential vulnerability for airway obstruction in a significant portion of humans lung, albeit to a significantly lesser degree than that predicted for rats assuming such a theoretical exposure was even possible.

## 4 Discussion

The MPPD model was used to evaluate differences between rats and humans in predicted regional airway deposition and retention of an organic pigment under exposure conditions obtained from an OECD guideline acute inhalation toxicity study. Although the test material is classified as a nanomaterial, current capabilities for generating atmospheres of low mg/L concentrations, including a limit test concentration of 5 mg/L, invariably resulted in agglomeration of the nano-sized particles, resulting in aerosols ranging in size from 2.01 to 2.74 microns MMAD in the acute inhalation case study in rats (Wittmer et al., 2021; Supplementary Table S1.1). This agglomeration behavior was also observed for other organic pigments in several dustiness measurements (EN 17199-4:2019-03), with peak aerodynamic diameters ranging from 1 to 5 μm with aerodynamic diameters increasing as aerosol concentrations increased. While Wessely et al. (2022) suggested that cascade impactors are not appropriate for characterizing particle sizes of low-density powders, due to the shearing of the fragile structure of the agglomerates during impactor measurements, the material used in the case study was not a low-density material, and no significant changes in particle sizes occurred in cascade impactor measurements taken over 2 h apart.

Under these exposure conditions, only 74%–78% of the 2.01- to 2.74-µm MMAD-sized atmospheric aerosols are predicted by MPPD to be inhalable by rats, while 100% is inhalable by humans. For nasal breathing under resting ventilation conditions, approximately 34% of respirable 2.74-µm MMAD aerosols deposited in the head of rats compared to 51% in humans (14% for oral breathing).

Since MPPD uses an empirical model for the head region (nose/mouth through the larynx), deposition within this region is not determined. However, previous simulations of comparable aerosols are available to estimate local deposition within the head regions of rats and humans by applying computational fluid particle dynamic (CFPD) modeling (Corley et al., 2021). Using CFPD modeling as a guide (see Supplementary Material), inhalable aerosols from the MPPD simulations for rats are predicted to deposit in the most anterior portions of the nose (nasal vestibule and wet squamous epithelium), followed by the most anterior portions of the respiratory and transitional epithelium of the nasal turbinates and maxilloturbinates, and larynx, the latter of which is consistent with the histopathological findings in a representative rat exposed to 5.212 mg/L in the acute inhalation study (Figure 1A). For humans, the CFPD analysis indicates that the nasal respiratory epithelium and the larynx also receive the highest deposited fractions following nasal breathing, whereas the mouth and larynx receive the highest deposition fractions following oral breathing. While each of these regions has high rates of aerosol clearance (ICRP, 2015), depending on the aerosol mode of action, the nasal respiratory epithelium (nasal breathing) and larynx (both nasal and oral breathing) could represent tissues of potential concern based on the local deposited dose in the head region of the MPPD model following exposures to high concentrations (5.212 mg/L) of aerosols of size 2.74 µm MMAD, unless cell-specific sensitivity factors across this region are known and suggest otherwise.

For the tracheobronchial region, MPPD provides both regional and generation- or airway-specific model analyses with comparable overall fractional depositions predicted for rats (∼5%) vs. humans (∼6%) for nasal breathing (∼16% for oral breathing). Overall, there is a generally higher fractional pulmonary deposition of 2.74 µm MMAD aerosols in humans (∼10–19%) than in rats (∼3%). There is also a shift toward significantly less deposition in the head and higher pulmonary deposition and deeper penetration into the pulmonary airways following oral breathing in humans.

Given the observation of significant airway obstruction in rats that died during or within 2 days following the 4-h exposure to the limit test concentration of 5 mg/L (5.212 mg/L actual) that were evaluated histologically (Figure 1; Wittmer et al., 2021), the calculations of potential airway obstruction from MPPD simulations in rats were consistent with the primary cause of death in that study. Not surprisingly, many tracheobronchial and pulmonary airways in rats were predicted to be vulnerable to obstruction and at risk for impairment of normal airflow and delivery to the gas-exchange region (Figures 4, 5). This prediction also supports findings of other similar chemicals in its class (Hofmann et al., 2018). Even after lowering the concentration from 5.212 mg/L, which caused all animals to die in the case study, to 2 mg/L, an alternative limit test concentration in the OECD guideline (not tested in the case study), or 1.084 mg/L (which did not cause lethality in the case study), a significant number of tracheobronchial and pulmonary airways might still be vulnerable to at least partial obstruction, albeit at a much lower percentage. These *in silico* results for the low-dose group are in accordance with the changed respiration pattern observed *in vivo* that persisted up to day 6 post-exposure and strongly indicated the presence of the previously described partial obstruction and altered airflow of the tracheobronchial and pulmonary airways. Given that these are the only effects observed in the case study (asphyxiation and altered respiration), these are not toxicity effects *per se* and raise questions about the classification of the potential human health risk of inhalation toxicity when labeling requirements are incongruent with the realities of guideline testing.

Clearly, humans would not be expected to tolerate exposures to the high aerosol concentrations of the pigment used in rats acute inhalation toxicity study even for a short period of time, much less for 4 h. However, with MPPD, *in silico* theoretical comparison of the potential for airway obstruction in rats is possible. In this case, differences across species in airway anatomy and physiology are major factors. Given the overall larger airway sizes and lower ventilation rate per kilogram body weight, it is not surprising that nearly 88% of all tracheobronchial and pulmonary airways (∼14.7 million) in humans following nasal breathing have deposited aerosol mass to airway diameter (obstruction) ratios <0.1 and ∼95% with ratios no more than 0.2. No airways were predicted to have ratios greater than 0.5 (Figure 6). By comparison, more than 75% of tracheobronchial and over 22% of pulmonary airways were predicted to have theoretical ratios >0.5 for deposited aerosol mass to airway diameters in rats, with nearly 8% of tracheobronchial and 0.6% of pulmonary airways with ratios >1.0 (Figure 5). While there is less chance for airway obstruction in humans, even under this extraordinary exposure condition, there still remains a concern given that nearly 97% (∼254,000) of tracheobronchial airways, primarily in generations 13–18, received deposited mass to airway diameter ratios between 0.3 and 0.4 (nasal breathing) or 0.4 and 0.5 (oral breathing), indicating a significant portion of the lung could still be vulnerable to physical obstruction if such a theoretical aerosol exposure was even possible in the real world.

There are acknowledged limitations to this study. Since MPPD cannot address the impact of particles on airflow (only the effect of airflow on particle transport), we assumed that particles deposited in each airway likely deposited in local hot spots (this typically occurs around bifurcations or when convective flow changes and impaction forces dominate (c.f. Anjivel and Asgharian, 1995; Corley et al., 2021)) and then coalesced together as each breath contributed additional particles to the same areas. The potential for further agglomeration over repeated breaths was considered likely given the propensity for the nanomaterial to agglomerate into micron-sized aerosols during aerosol generation. Assuming the aerosol mass was spherical, the resulting aerosol diameters could be compared with the diameter of each corresponding airway as a predictor of airway obstruction potential. One aspect to this assumption is that even with a calculated full airway obstruction, without a two-way coupled airflow–particle interaction, MPPD will predict that particles still penetrate deeper into the lung periphery as if no upstream obstruction exists. This in effect reduces the impact of the calculations as we ignore the fact that airways and respiratory regions beyond an obstruction are no longer ventilated, and more particles build up near each obstruction with each subsequent breath. This is one justification for the importance of histopathology observations and *in silico* predictions of obstruction in the tracheobronchial region of conducting airways that ventilate the pulmonary/alveolar regions.

The other potential limitation is that the current analysis focuses on total deposition over 4 h of exposure and ignores the potential for particle clearance. When particle clearance was factored into simulations, no pulmonary clearance of particles (primarily macrophages) was predicted to occur within the 4-h exposure in either rats or humans. In the tracheobronchial region, mucociliary clearance, which is normally faster in rats and humans, could result in ∼22% of deposited mass retained at the end of the exposure in rats, while ∼81% would be retained in humans based on comparative simulations at the limit test exposure concentration. However, this means that mucociliary clearance would be unaffected by such an exposure. Furthermore, it is theoretically possible, if not probable at such high exposures, that as the mucociliary escalator transports deposited aerosol mass from distal generations toward the proximal upper conducting airways, more particles can agglomerate and impede mucociliary transport as airways come together. For repeated exposure or longer-term studies, incorporating aerosol clearance would be important, but for an acute 4-h exposure, as in the current analysis, deposited dose is a reasonable dose metric.

In addition to direct evidence from histopathology, there is also supporting, indirect information that the coalescing of deposited material likely occurs for the specific organic nano-sized pigment evaluated in the acute inhalation study by Wittmer et al. (2021). The first is that all efforts to generate test atmospheres from the nanomaterial pigment up to concentrations of 1–5 mg/L still resulted in aerosol sizes in the micrometer range. In addition, *in vitro* studies measuring the hydrophobicity of a series of nano-sized organic pigments showed that hydrophilic materials spread out on the surface of an adhesive, transparent tape, whereas hydrophobic materials “clumped up” into droplet shapes using a drop shape analyzer, one of several measured properties that were associated with acute inhalation lethality through asphyxiation (Hofmann et al., 2018). Even with these acknowledged limitations, taken together, this *in silico* approach serves as a simplified, yet useful, comparison for materials with properties similar to the organic, nano-sized pigment evaluated in the acute inhalation study.

In keeping with the decades-old 3R principles for humane use of animals in research (replace, reduce, and refine; Russell and Burch, 1959), OECD test guidelines for acute inhalation studies were originally designed to minimize the number of animals to meet the scientific objectives of the study. Furthermore, limit concentrations were established to provide an upper bound on exposures for materials that are relatively non-toxic. If animals survive and recover from 4-h exposures to the limit concentration, no further testing is required, and animal use is minimized. While OECD recognizes that a limit test of 5 mg/L is challenging for many aerosols and can exceed real-world exposures, a lower limit of 2 mg/L may be justified, which prompted the use of this exposure concentration in the case study. This (2 mg/L) is also the limit test concentration used in the U.S. EPA acute inhalation guidelines (EPA, 1996). However, testing of insoluble, nano-sized dust particles for acute toxicity according to the current OECD guidelines continues to present several ethical, regulatory, and technical problems even for a 2 mg/L alternative limit exposure based on this *in silico* analysis.

Although an update of the GHS labeling criteria for acute inhalation toxicity and harmonization with OECD guidance documents 403 and 39 for nanomaterials is time-consuming and changes in an international regulation even more so, the results from this study and those of other materials in this class suggest that a thorough update is overdue. The technical performance and the obstacles in the *in vivo* experiment when testing insoluble dust particles are also subjects for further investigations, and the results should be incorporated in updated guidelines reflecting nanomaterial properties. Moreover, the re-worked guidance documents and the classification and labeling regulation must be harmonized with testing guidelines to enable a regulatory assessment for this category of testing materials. For the time being, MPPD simulations of airway deposition at high exposure concentrations between rats and humans, in addition to estimating the extent to which airways may be vulnerable to obstruction, form a reasonable basis for an interim assessment on potential health hazards for certain classes of nanomaterials and their relevance, or lack thereof, for humans.

## Data Availability

The original contributions presented in the study are included in the article/Supplementary Material; further inquiries can be directed to the corresponding author.
