# Supplementary material for: Refinement of the acute inhalation limit test for inert, nano-sized dusts by an in silico dosimetry-based evaluation: case study for the dissolution of a regulatory dilemma
Source: Front Toxicol. 2023 Dec 5;5:1258861. doi: 10.3389/ftox.2023.1258861 (PMC10729606; doi:10.3389/ftox.2023.1258861)
Supplement: Supplementary file 1 [file DataSheet1.docx]

# Refinement of the Acute Inhalation Limit Test for Inert, Nano-sized Dusts by an In silico Dosimetry-Based Evaluation. Case study for the dissolution of a regulatory dilemma

Heidi Stratmann^1^, Lan Ma-Hock^2^, Simone Tangemann^2^ and Richard A. Corley^3^

^1^Colors & Effects Switzerland AG

Efringerstrasse 32

4057 Basel, Switzerland

^2^Experimental Toxicology and Ecology

BASF SE

67056, Ludwigshafen, Germany

^3^Greek Creek Toxicokinetics Consulting, LLC

4659 W. Deer Path Dr.

Boise, Idaho 83714, USA

**CONTENTS**

1. Summary of acute inhalation toxicity study with copper phthalocyanine pigment
2. Theoretical distribution of aerosols deposited within the head regions of the rat and human based upon CFPD modeling of similar exposures

## ACUTE INHALATION TOXICITY STUDY WITH COPPER PHTHALOCYANINE PIGMENT (Wittmer et al., 2021)

### Background

Organic pigments are colored materials which are practically insoluble in the intended application medium in which they are incorporated (Zweifel 2001; Christie 2014). They are widely used in the coloring of inks, paints, rubber, and plastic products. Typically, the chemical constituents of organic pigments are planar or nearly planar molecules within the crystal lattice; phthalocyanine pigments serve hereby as prime examples (Barrow 2002). Blue copper phthalocyanines are copper(II) complexes of tetraazatetrabenzoporphine forming a planar and completely conjugated ring system which exhibits exceptional stability. All crystal modifications of the molecule (α, β, γ, δ, ε) are arranged like rolls of coins varying in the angle of the stacked molecules to the longitudinal axis. The largely non-polar molecule in combination with the structural arrangement gives little leeway for specific interaction with the surrounding medium being the root cause of the very low hydrophilicity of these pigments (Hunger and Schmidt, 2018). Milling and kneading is one of the finishing techniques of the crude pigment and crucial to control on the one hand the intended crystal modification and determines on the other hand particle size and shape. Depending on the respective finishing step, the pigment particle shape is irregular at low aspect ratio without crystalline facets, and may belong to spheroidal, platelet or elongated shape categories. Finishing of crude copper phthalocyanine pigments for example leads to generation of particles below 100 nm classified as nano materials according to the *recommendation of the European Commission* (European Commission 2011). While the size of the primary particles is in the nano range, currently available methods for generating aerosols from this class of materials at a limit test concentration unavoidably result in aerosols in the micron range due to agglomeration. This agglomeration behavior was observed for organic pigments in several dustiness measurements (EN 17199-4:2019-03) with peak aerodynamic diameters ranging from 1-5 μm.

### Test material

Low chlorinated copper, [29H,31H-phthalocyaninato(2-)-N29,N30,N31,N32]-, wherein the number of chlorines is more than or equal to 0 and less than or equal to 7, CAS 27614-71-7 was used as the test item. The sample was handled as blue powder having a purity of >99%. The tested compound was manufactured by Sun Chemical Colors & Effects GmbH.

### Methods

The acute inhalation study was performed in male and female Wistar rats according to OECD-Guideline method 403 (acute inhalation toxicity) with full GLP compliance. The laboratories of BASF's Experimental Toxicology and Ecology, where the study was performed, are AAALAC-certified. All procedures for animal care and exposure were conducted under the rule of the German Animal Welfare Act (1998). This study was approved by the local authorizing agency for animal experiments (Landesuntersuchungsamt Rheinland-Pfalz (Koblenz, Germany)) as referenced by the approval number 23 177-07/G 17-3-063.

Dust aerosols were generated by means of dust generators using compressed air into a mixing stage, mixed with conditioned air and passed into the inhalation systems. The desired concentration was achieved by varying the piston feed, the brush rotation rate and amount of conditioned air and exhaust air.

The atmospheric concentration was determined hourly by gravimetrical measurements of air samples taken adjacent to the animals´ breathing zone (sampling velocity 1.25 m/s). A defined volume of air was sampled by vacuum pump through a binder-free glass-fiber filter (Macherey-Nagel, MN 85/90 BF, 4.7 cm diameter).

Cascade impactor measurements were performed using stack sampler Marple 298 (New Star Environmental, Inc., Roswell, Georgia 30075, USA). Pre-weighed metal collecting discs and a backup particle filter were placed into the cascade impactor. Two samples per concentration group were taken at a sampling velocity of 1.25 m/s from the breathing zones of the animals. After sampling the collecting discs and backup filter were weighed again and the weight of test substance, collected at each stage, calculated by difference. The amount for each stage was used to determine the cumulative amount below each cut-off point size. In this way, the proportion (%) of aerosol less than 17.3, 11.9, 7.9, 4.8, 2.8, 1.3, 0.7 and 0.4 μm was calculated. The mass median aerodynamic diameter (MMAD) and geometric standard deviation (GSD) was calculated on the basis of mathematical method described OECD guidance document No. 39.

Male and female Wistar rats were obtained from Charles River Laboratories, Sandhofer Weg, Sulzfeld, Germany (7 weeks of age, strain Crl:WI (Han)), and were allowed free access to mouse/rat laboratory diet (Provimi Kliba SA, Kaiseraugst, Switzerland) and water. The animals were group-housed (5 animals/cage) in polysulfone cages in accommodation maintained at 20 to 24°C, with a relative humidity of 30 to 70%, a light/dark cycle of 06.00 to 18.00 h light and 18.00 to 06.00 h dark and were allowed to acclimatize to these conditions for approximately two weeks before commencement of the study.

Groups of 5 male and female Wistar rats per concentration were nose-only exposed to respirable dusts for 4 hours. The animals were observed for any signs of toxicity during a total of fourteen post exposure days. Body weights were determined on the day of exposure and on days 1, 3 7 and 14. Animals that died prematurely underwent gross necropsy as soon as possible, as well as all surviving animals at the end of the post-exposure period. The exposure was performed in staggered manner. The first exposure was performed at 5 mg/L, which is the limit concentration of the OECD guideline 403. This high concentration was tested for the following two reasons. First, no specific toxicity was observed in an oral acute study, in skin and eye irritation tests or skin sensitization assay. Neither were there any indication for any toxicity in all available studies on this substance or analogues together (e.g. OECD 408 or 421). Second, dust atmosphere with high fraction of respirable particles could be generated at this high concentration. As animals died at this high concentration of 5 mg/L, a second concentration was targeted at 1 mg/L which was the threshold concentration for category III and IV.

The prematurely death was unexpected for this test substance, the respiratory tract of representative animals (one male and two females) were examined histopathologically considering the recommendation for such examinations in the respective OECD guideline for repeated dose inhalation studies.

1. The lungs were removed intact, weighed and instilled with 4 % buffered formalin at a pressure of 20–30 cm of water to ensure that lung structure was maintained. Each of the 5 lung lobes representing different regions of the lobe were examined by light microscopy.
2. For nasal cavity, four levels of the nasopharyngeal tissues were examined, one of which includes the nasopharyngeal duct.
3. Three levels of the larynx were examined, and one of these levels includes the base of the epiglottis.
4. Two levels of the trachea were examined including one longitudinal section through the carina of the bifurcation of extrapulmonary bronchi and one transverse section.

### Results

*Exposure Conditions*

**Supplemental Table S1.1.** Analytical exposure concentrations and particle size analysis.

| **Test group** | **Mean Concentration (mg/L)** | **St. Dev** | **Nominal concentration (mg/L)** | **MMAD (µm)** | **GSD** |
| --- | --- | --- | --- | --- | --- |
| 1 | 1.084 | 0.118 | 2.3 | 2.01 | 3.0 |
| 2 | 5.212 | 0.216 | 7.5 | 2.74 | 2.8 |

*Clinical signs of toxicity, including mortality*

The duration of the clinical finding of test group 2 was shorter than in test group 1, because all animals died before day 2 post-exposure.

**Supplemental Table S1.2:** Duration of signs

|  | **Test group 1**  **(1.084 mg/L)** | | **Test group 2**  **(5.212 mg/L)** | |
| --- | --- | --- | --- | --- |
|  | **Male** | **Female** | **Male** | **Female** |
| Lethality (number of animals) | 0/5 | 0/5 | 5/5 | 5/5 |
| Time of sound dead | - | - | h3, d1 | d0 - d2 |
| Activity/behavior, attention reduced | - | - | d0 | d0-d1 |
| Feces, no feces | - | - | - | d1 |
| Feces, discolored feces, substance like | d1 | d1 | - | - |
| Fur discolored, substance like | d2-d11 | d2-d14 | d0 | d0-d1 |
| Piloerection | d0-d1 | d0-d1 | d0 | d0-d1 |
| Fur, substance-contaminated | d0-d1 | d0-d1 | d0 | d0 |
| Respiration, abdominal | - | d0 -d4 | d0 | d0-d1 |
| Respiration, accelerated | h1-d6 | h1-d5 | h1-h2 | h1-h2 |
| Respiration, depressed | - | - | h3-h4 | h3-h4 |
| Respiration, intermittent | d5-d6 | d0-d1,  d5-d6 | - | - |
| Respiration, sounds | d2-d4 | - | - | - |

*Necropsy findings*

**Supplemental Table S1.3:** Animals that died during the study period

| **Findings** | **test group 2 (5.2 mg/L)** |
| --- | --- |
| Number of animals | 5 males + 5 females |
| Lung:  many black foci in all lobes (Æ 4 mm), surface sunken | 1 male + 3 females |
| Lung:  many black foci in all lobes (Æ 8 mm), surface sunken | 4 males + 2 females |
| Stomach:  blue discoloration of the content | 4 males + 2 females |
| Trachea:  blue deposition | 4 males + 2 females |

For further evaluation, histopathological examinations of the respiratory tract (nasal cavity, larynx, pharynx, trachea and lung) from animals Nos. 793, 798 and 800 (test group 2) were performed. The following findings were noted:

S**upplemental Table S1.4:** Histopathological findings

|  | Animal No.: | | |
| --- | --- | --- | --- |
|  | 793 | 798 | 800 |
| **Lung** |  |  |  |
| Bronchi, bronchioles and terminal bronchioles contain large amount of blue pigment | x | - | - |
| Bronchi, bronchioles and terminal bronchioles contain large amount of blue pigment, which obstructs the lumen | - | x | - |
| Bronchi, bronchioles and terminal bronchioles contain large amount of blue pigment, which obstructs the lumen, emphysema | - | - | x |
|  |  |  |  |
| **Trachea** |  |  |  |
| Dilation, contains blue pigment | x | x | x |
|  |  |  |  |
| **Larynx** |  |  |  |
| Level I Obstructed by blue pigment | x | - | x |
| Level I Contains large amounts of blue pigment | - | x | - |
| Level II Obstructed by blue pigment | x | - | x |
| Level II Contains large amounts of blue pigment, edema | - | x | - |
| Level III Contains large amounts of blue pigment | x | - | x |
| Level III Contains small amounts of blue pigment | - | x | - |
|  |  |  |  |
| **Nasal cavity** |  |  |  |
| Level I Contains small amounts of blue pigment | x | x | x |
| Level II Contains small amounts of blue pigment | x | - | x |
| Level II Contains moderate amounts of blue pigment |  | x |  |
| Level III Contains small amounts of blue pigment | x | x | x |
| Level IV Contains small amounts of blue pigment | x | x | x |

The histopathological findings in the lung, the trachea and the larynx of animal no. 793, 798 and 800 indicate an airway obstruction caused by the inhaled blue pigment as cause of death.

### References

Barrow, M. J., Christie, R. M., & Monteith, J. E. (2002). The crystal and molecular structures of three diarylide yellow pigments, CI Pigments Yellow 13, 14 and 63. Dyes and pigments, 55(2-3), 79-89.

Christie, R. (2014). Colour chemistry. Royal society of chemistry.

Hunger, K., & Schmidt, M. U. (2018) Industrial Organic Pigments: Production, Crystal Structures, Properties, Applications, Fourth, Completely Revised Edition.

European Commission. (2011). Commission recommendation of 18 October 2011 on the definition of nanomaterial. Off. J. Eur. Union, 275, 38.

Zweifel, H., Maier, R. D., Schiller, M., & Amos, S. E. (2001). Plastics additives handbook

Wittmer E, Tangermann, S., and Landsiedel, R. 2021. Low chlorinated copper, [29h,31h-phthalocyaninato(2-)-n29,n30,n31,n32]-, wherein the number of chlorines is more than or equal to 0 and less than or equal to 7. Acute inhalation toxicity study in wistar rats. 4-hour dust exposure (nose only). Final report. Experimental Toxcology and Ecology, BASF SE.

## THEORETICAL DISTRIBUTIONS OF AEROSOL DEPOSITION WITHIN THE HEAD REGION OF THE RAT AND HUMAN

The MPPD model uses a simple empirical model to determine aerosol deposition in the head region (nose through larynx) of rats without differentiating local regions within the head that are common target sites for deposition and potential toxicity in inhalation studies due to their unique airway anatomy and breathing patterns. However, previous computational fluid particle dynamics (CFPD) model provided site-specific deposition patterns in the upper conducting airways of the Sprague Dawley rat with comparable aerosol exposures that can inform the results from the MPPD simulations as both the CFPD and MPPD models utilized the same source of CT-derived airway geometry data (Corley et al., 2021).

**Supplemental Figure S2.1.** Annotated upper airway surfaces encompassed by the head regions of MPPD models along with sites of particle deposition in the (A) rat and (B) human CFPD models following exposure to 2.72 µm MMAD aerosols at a concentration of 4.03 mg/l (from Figure 3 of Corley et al., 2021).

(A)


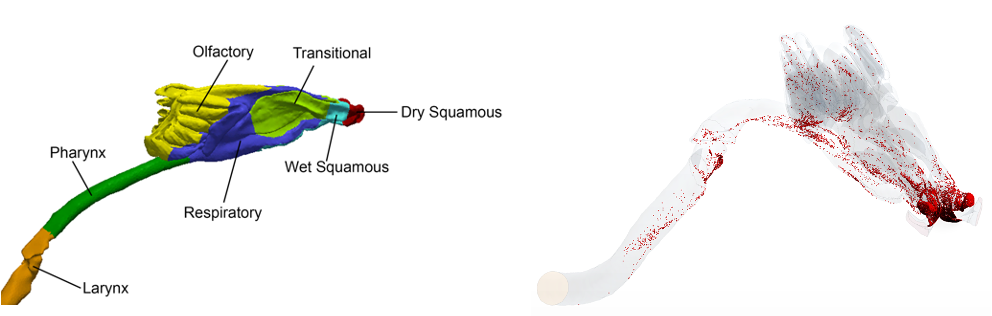


(B)

**
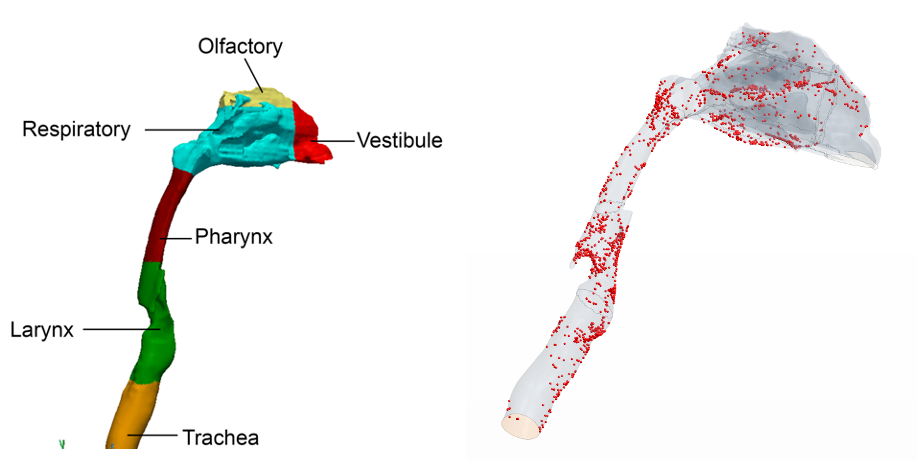
**

### **Theoretical Distribution of Aerosol Deposition Within the Head Region of the Rat.**

In the CFPD rat model, regional deposition fractions in the nasal vestibule (dry squamous epithelium); wet squamous, respiratory, transitional, and olfactory epithelium; and the nasopharynx and larynx regions were determined for a single breath of exposure to 2.72 µm MMAD aerosols at 4.03 mg/L air concentration. To simplify the comparison, MPPD used the same MMADs and GSD as the CFPD simulations, not the MMADs and GSDs from the acute toxicity study. The main differences between the simulations were with the simplified one-dimensional airway geometry used in the empirical MPPD head model vs. the actual three-dimensional airway geometry used in the CFPD model and the respective breathing profiles used in each simulation (CFPD: 217 ml minute volume at 100 bpm; MPPD: 353 ml minute volume, 166 bpm). In addition, the use of an inhalability correction in MPPD which reduced the inhalable fraction to 0.79, but not with the CFPD simulation (no inhalability adjustment needed as particles were introduced directly into the lumen of the nasal inlet based upon local airflow rate) was largely responsible for differences in total inhaled fractions deposited in the head regions between the two models (CFPD: 0.59 vs. MPPD: 0.35). Regardless, estimated fractional deposition of inhalable aerosols within each head region of the CFPD model could still be used to normalize the total fraction deposited from the MMPD head simulations to corresponding theoretical regions within the Asymmetric Sprague Dawley rat models (**Supplemental Table S2.1A**). Thus, most inhalable aerosols from the MPPD simulations are predicted to deposit in the anterior-most portions of the nose (nasal vestibule and wet squamous epithelium) followed by the anterior portions of respiratory and transitional epithelium of the nasoturbinates and maxilloturbinates, and larynx. Of these tissues, the anterior portions of respiratory and transitional epithelium of the nasoturbinates and maxilloturbinates and the larynx are common target sites for inhaled materials that produce local inflammation, irritation, or cytotoxicity in the head region. Since the Asymmetric Sprague Dawley rat models utilized the same empirical head model, results for the two models were essentially the same.

**Supplemental Table S2.1.** Theoretical distributions of the fractions of deposited mass estimated for the head region of the (A) rat and (B) human MPPD simulations based upon published computational fluid particle dynamics (CFPD) simulations with comparable aerosols (Corley et al., 2001).

1. Rat

| **From Table 2, Corley et al. (2021)** | | | **Normalized Deposition in MPPD Head Region Asymmetric SD Rat** | | | |
| --- | --- | --- | --- | --- | --- | --- |
| **Airway Region** | **Surf. Area (cm^2^)** | **Dep. Fract.** | **Est. Dep. Fract.** | **Est. Dep Mass Rate (µg/Breath)** | **Est. Dep Mass Rate (µg/min)** | **Est. Total Dep Mass 4-hr (µg)** |
| Vestibule, Dry Squamous | 0.45 | 0.5310 | 0.2915 | 3.10 | 514.3 | 123,439.0 |
| Wet Squamous | 0.63 | 0.0483 | 0.0265 | 0.28 | 46.8 | 11,230.0 |
| Respiratory | 5.69 | 0.0010 | 0.0005 | 0.01 | 1.0 | 228.2 |
| Transitional | 2.16 | 0.0016 | 0.0009 | 0.01 | 1.6 | 374.4 |
| Olfactory | 6.75 | 0.0002 | 0.0001 | 0.00 | 0.2 | 46.9 |
| Pharynx | 1.32 | 0.0002 | 0.0001 | 0.00 | 0.2 | 44.3 |
| Larynx | 0.38 | 0.0032 | 0.0018 | 0.02 | 3.1 | 741.2 |
| **Total** | **17.39** | **0.5855** | **0.3214** | **3.4163** | **567.1** | **136,104.0** |

1. Human

| **From Tables 4 and 5, Corley et al. (2021)** | | | **Normalized Deposition in MPPD Head Region** | | | |
| --- | --- | --- | --- | --- | --- | --- |
| **Nose Breathing** | | | **Nose Breathing (Resting)** | | | |
| **Airway Region** | **Surf. Area (cm^2^)** | **Dep. Fract. (a)** | **Est. Dep. Fract.** | **Est. Dep Mass Rate (µg/Breath)** | **Est. Dep Mass Rate (µg/min)** | **Est. Total Dep Mass 4-hr (µg)** |
| Vestibule | 33.0 | 0.00034 | 0.0269 | 83.9 | 1,006.9 | 241,656.6 |
| Respiratory | 181.4 | 0.00359 | 0.2849 | 890.3 | 10,683.0 | 2,563,930.8 |
| Olfactory | 21.3 | 0.00033 | 0.0258 | 80.6 | 966.7 | 232,013.1 |
| Pharynx | 29.1 | 0.00048 | 0.0381 | 119.1 | 1,429.5 | 343,068.4 |
| Larynx | 33.0 | 0.00164 | 0.1297 | 405.3 | 4,863.9 | 1,167,331.1 |
| **Total** | **297.732** | **0.00637** | **0.5054** | **1,579.2** | **18,950.0** | **4,548,000.0** |
| **Oral Breathing** | | | **Oral Breathing** | | | |
| Mouth | 77.8 | 0.00597 | 0.0226 | 70.58 | 846.9 | 203,263.1 |
| Oropharynx | 44.0 | 0.00070 | 0.0026 | 8.23 | 98.7 | 23,693.1 |
| Larynx | 33.9 | 0.00155 | 0.0059 | 18.36 | 220.3 | 52,883.8 |
| Total | 155.6 | 0.00822 | 0.0311 | 97.17 | 1,166.0 | 279,840.0 |

1. Interpolated from deposition fractions in airway regions for 2.75 µm aerosols from 1 and 3 µm aerosol simulations (Tables 4 and 5, Corley et al. (2021)).

### **Theoretical Distribution of Aerosol Deposition Within the Head Region of the Human.**

As with the rat, the theoretical distributions of airway deposition across cell type or anatomic region in the empirical head region of the Symmetric Yeh and Schum MPPD simulations for resting oral and nasal breathing were calculated using published CFPD simulations and are summarized in **Supplemental Table S2.1B** (Corley et al., 2021). Based upon these calculations, the respiratory epithelium and the larynx are predicted to receive the greatest deposited fractions and cumulative deposited masses of aerosols following nasal breathing vs. the mouth and larynx following oral breathing. While each of these regions have high rates of aerosol clearance (ICRP, 2015), depending upon the aerosol toxicity mode of action, the nasal respiratory epithelium (nasal breathing) and larynx (both nasal and oral breathing) could represent tissues of potential concern just based upon local deposited dose in the head region of MPPD following exposures to such high concentrations of aerosols of this size (5 mg/L, 2.75 µm) unless cell-specific sensitivity factors across this region are known and suggest otherwise.

### References

Corley, R.A., Kuprat, A.P., Suffield, S., Kabilan, S., and Hinderliter, P., Yugulis, K. and Ramanarayanan, T.S. (2021). New approach methodology for assessing inhalation risks of a contanct respiratory cytotoxicant: computational fluid-particle dynamic-based aerosol dosimetry modeling for cross-species and in vitro comparisons. *Toxicol*. *Sci.* **182(2),** 243-259.

ICRP (International Commission on Radiological Protection). (2015). Occupational Intakes of Radionuclides: Part 1. ICRP Publication 130. Ann. ICRP 44(2). Sage
